# Sibjotang Increases Atrial Natriuretic Peptide Secretion in Beating Rabbit Atria

**DOI:** 10.1155/2015/268643

**Published:** 2015-10-01

**Authors:** Oh Jeong Kwon, Hyun Cheol Oh, Yun Jung Lee, Hye Yoom Kim, Rui Tan, Dae Gill Kang, Ho Sub Lee

**Affiliations:** ^1^College of Oriental Medicine and Professional Graduate School of Oriental Medicine, Wonkwang University, Shinyong-dong, Iksan, Jeonbuk 570-749, Republic of Korea; ^2^Hanbang Body-Fluid Research Center, Wonkwang University, Shinyong-dong, Iksan, Jeonbuk 570-749, Republic of Korea; ^3^College of Pharmacy, Wonkwang University, Iksan 570-749, Republic of Korea; ^4^Brain Korea (BK) 21 Plus Team, Professional Graduate School of Oriental Medicine, Wonkwang University, Iksan, Jeonbuk 540-749, Republic of Korea

## Abstract

Sibjotang (Shizaotang), traditional herbal medicine formula, which was first documented in the Shanghanlun, has long been prescribed for the treatment of impairment of the body fluid homeostasis. The purpose of the present study was to identify the effects of Sibjotang on the secretion of a cardiac hormone, atrial natriuretic peptide (ANP), one of the main hormones involved in the regulation of the body fluid and blood pressure homeostasis. Water extract of Sibjotang increased ANP secretion concomitantly with an increase in atrial dynamics in a concentration-dependent manner. Sibjotang-induced increase in ANP secretion and positive inotropic effect were attenuated by GO6976 and LY333531, selective inhibitors of conventional protein kinase C, but not Rottlerin, an inhibitor of novel PKC_*δ*_. Similarly to the effect of Sibjotang, extracts of components of Sibjotang, *Euphorbia kansui*, and *Daphne genkwa*, but not *Euphorbia pekinensis* and *Ziziphus jujuba*, increased ANP secretion and atrial dynamics. Ingredients of Sibjotang, apigenin, rosmarinic acid, and salvianolic acid B decreased ANP secretion and atrial dynamics. These findings suggest that Sibjotang increases ANP secretion and atrial dynamics via activation of conventional protein kinase C signaling. This finding provides experimental evidence for the rationale in the use of Sibjotang in the treatment of impairment of the regulation of body fluid and blood pressure homeostasis.

## 1. Introduction

Regulation of body fluid homeostasis is considered to be important to maintain healthy body function. Both humoral and autonomic nervous systems are involved in the regulation of body fluid homeostasis. Cardiac atria are the site of sensing the change of fluid volume and responding to correct the change via the secretion of atrial natriuretic peptide (ANP, atrial natriuretic hormone), a member of the family of cardiac natriuretic hormones. ANP is synthesized and stored in and released from the cardiac myocytes. The natriuretic peptide receptors coupled with guanylyl cyclase or nonguanylyl cyclase signaling are widely distributed throughout the whole body including the cardiorenal system [1, 2]. The natriuretic peptide system is classically involved in the regulation of body fluid volume and blood pressure homeostasis via cardiorenal communication. ANP increases urinary excretion of water and electrolytes and decreases blood pressure via activation of natriuretic peptide receptors coupled with guanylyl cyclase-cGMP signaling. In addition to the classical effects, the ANP system is also involved in the antioxidative, anti-ischemic, anti-inflammatory, and antiproliferative functions [3–5]. Furthermore, the ANP system is involved in the regulation of energy homeostasis including lipid mobilization [6, 7]. Plasma ANP levels are primarily controlled by the rate of secretion of ANP from the atria. ANP secretion from the atria is under the control of atrial volume changes, adrenergic and muscarinic influences, and hormones [8–10]. Intracellular second messengers including cGMP, cAMP, Ca^2+^, and K^+^ channels are involved in the regulation of ANP secretion [9, 11]. Activation of protein kinase C (PKC) is also known to increase ANP secretion [12].

Sibjotang (Jyusoto in Japanese, Shizaotang in Chinese), traditional herbal medicine formula, has been prescribed for the treatment of impairment of the regulation of body fluid homeostasis such as edema in Asian countries. The formula is composed of four component herbal medicines:* Euphorbia kansui*,* Euphorbia pekinensis*,* Daphne genkwa*, and* Ziziphus jujube*. Sibjotang was first written in Shanghanlun, an old medical book of China. Although the formula has long been prescribed, the mechanism of action of the formula has not yet been identified. The purpose of the present study was to identify the acute and direct effects of water extract of Sibjotang (SJ) on the secretory and contractile function of the cardiac atria and the regulation of ANP secretion in perfused beating rabbit atria.

## 2. Methods and Materials

### 2.1. Reagents

GO6976 (GO), LY333531 (LY), Rottlerin (Rott), rosmarinic acid, and apigenin were purchased from Sigma Chemical Co. (Yongin, Korea). Salvianolic acid B was purchased from Santa Cruz (Santa Cruz, CA, USA). Stock solutions of GO, LY, Rott, and apigenin were dissolved in dimethyl sulfoxide (DMSO). Salvianolic acid B and rosmarinic acid were dissolved in distilled water. The final concentration of DMSO was less than 0.1%.

### 2.2. Preparation of a Water Extract from Sibjotang (SJ)

Sibjotang is composed of four medicinal herbs,* Euphorbia kansui* (37.5 g),* Euphorbia pekinensis* (37.5 g),* Daphne genkwa* (37.5 g), and* Ziziphus jujube* (85 g). The above medicinal herbs were purchased from the Herbal Medicine Cooperative Association of Iksan, Jeonbuk. Herbarium voucher specimens of SJ (HBG132) were prepared and deposited in the Herbarium of the Professional Graduate School of Oriental Medicine, Wonkwang University, Iksan, Korea.* Euphorbia kansui*,* Euphorbia pekinensis*,* Daphne genkwa*, and* Ziziphus jujube* were prepared and deposited in the same sites, too. Mixture of the dried herbs (197.5 g) was extracted with 2,000 mL of boiling distilled water for 2 h. The extract was filtered through Whatman number 5 filter paper and concentrated using rotary evaporator. Freeze-dried extract (SJ, 44 g) was obtained and stored at desiccated deep freezer until used. Extraction of component herbs of SJ was performed similarly.

### 2.3. Atrial Preparation

Male New Zealand White rabbits (~1.8 kg) were used. The study was carried out with the approval of the Institutional Animal Care and Utilization Committee for Medical Science of Wonkwang University. Rabbits were anesthetized with Ketamine (50 mg/kg, iv). After anesthesia, rabbits were exsanguinated and the heart was dissected and remaining blood was washed in oxygen-containing saline solution. The left atrium was isolated. Preparation of perfused beating atria was followed by the method previously described [13]. A transparent cannula containing 2 smaller polyethylene tubings inside the cannula was introduced into the atrial lumen. Transparent cannula has the scale outside. A platinum wire was located inside the cannula allowing transmural electrical field stimulation. One polyethylene tubing within the cannula was used to infuse perfusion fluid (HEPES buffer, 1 mL/min) and the other one was used to measure the changes in pressure inside the atrium. A cannulated atrium immediately transferred to an organ chamber maintained at 34°C and stimulated by electrical stimulation (1.3 Hz, 0.3–0.5 ms, 30–40 V). The organ chamber contained 3 mL buffer solution which is the same composition as the perfusion fluid and the chamber was supplied with oxygen by silicone tubing inside. The composition of the HEPES buffer follows (mM): 118 NaCl, 4.7 KCl, 2.5 CaCl_2_, 1.2 MgCl_2_, 25 NaHCO_3_, 10 glucose, 10 HEPES, and 0.1% bovine serum albumin (pH 7.4). Atrial pressure was measured continuously by PowerLab/8SP (model ML 785, AD Instruments, Australia). The atrial stroke volume was measured by measuring the change of the diastolic and systolic levels of the water column of the transparent cannula. Perfusate was collected at every 2 min in a test tube in ice-cold water bath.

### 2.4. Experimental Protocols

**Figure 1:**
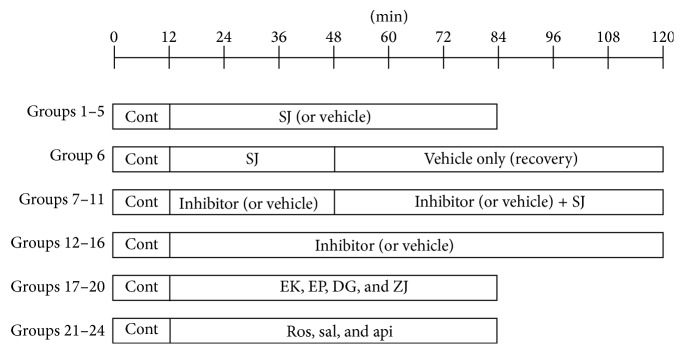
Protocols for present experiments. Atria were paced 1.3 Hz. The values (means of two fractions) obtained before and after the addiction of vehicle (LY333531, GO6976, Rottlerin). Cont: control; SJ: Sibjotang; LY: LY333531; GO: GO697; Rott: Rottlerin; EK,* Euphorbia kansui*; EP:* Euphorbia pekinensis*; DG:* Daphne genkwa*; ZJ:* Ziziphus jujube*; ROS: rosmarinic acid; SAL: salvianolic acid B; API: apigenin. See Section 2.4 for details.

The beating atria were perfused with buffer solution for 40 min to stabilize pulse pressure, stroke volume, and ANP secretion. The perfusate was collected serially at 2 min intervals at 4°C for analysis. Experiments were carried out using 24 groups of atria (Figure 1) to answer the following questions.

#### 2.4.1. Does SJ Affect Atrial ANP Release?

To identify the effects of SJ, 12 min of control was followed by 72 min of SJ (group 1, 0 *μ*g/mL, *n* = 6; group 2, 60 *μ*g/mL, *n* = 8; group 3, 100 *μ*g/mL, *n* = 8; group 4, 300 *μ*g/mL, *n* = 5; and group 5, 1,000 *μ*g/mL, *n* = 5). To test the recovery from the effects of SJ, 36 min of SJ infusion was followed by 72 min of buffer only (group 6, 1,000 *μ*g/mL, *n* = 4).

#### 2.4.2. Are the SJ-Induced Changes in ANP Release Related to PKC Pathway?

In another series of experiments to identify the mechanisms involved, 12 min of control was followed by an inhibitor of PKC_*α*_, PKC_*β*_, or PKC_*δ*_ for 36 min and then SJ in the presence of previous agent for 72 min (group 7, DMSO 0.1% + SJ 100 *μ*g/mL, *n* = 9; group 8, GO6976 (GO) 100 nM + SJ 100 *μ*g/mL, *n* = 9; group 9, LY333531 (LY) 30 nM + SJ 100 *μ*g/mL, *n* = 12; group 10, LY 100 nM + SJ 100 *μ*g/mL, *n* = 8; group 11, Rottlerin (Rott) 1,000 nM + SJ 100 *μ*g/mL, *n* = 8; group 12, DMSO 0.1% + vehicle, *n* = 8; group 13, GO 100 nM + vehicle, *n* = 5; group 14, LY 30 nM + vehicle, *n* = 6; group 15, LY 100 nM + vehicle, *n* = 5; and group 16, Rott 1,000 nM + vehicle, *n* = 6).

#### 2.4.3. Do Components and Each Herb Affect Atrial ANP Release?

In another series of experiments, effects of extract of the component herbs of the formula,* Euphorbia kansui*,* Euphorbia pekinensis*,* Daphne genkwa*, and* Ziziphus jujube*, were tested separately (group 1, vehicle control, *n* = 6; group 17,* Euphorbia kansui* 200 *μ*g/mL, *n* = 7; group 18,* Daphne genkwa* 200 *μ*g/mL, *n* = 7; group 19,* Euphorbia pekinensis* 200 *μ*g/mL, *n* = 7; and group 20,* Ziziphus jujube* 400 *μ*g/mL, *n* = 7). In another series of experiments, effects of apigenin (api), rosmarinic acid (ros), and salvianolic acid B (sal) were tested (data not shown). Twelve min of control was followed by 72 min of water extract of component herbs or vehicle (group 1, control for rosmarinic acid and salvianolic acid B, *n* = 6; group 21, control for apigenin, DMSO 0.1%, *n* = 5; group 22, apigenin 10 nM, *n* = 5; group 23, rosmarinic acid, 1.0 *μ*M, *n* = 5; and group 24, salvianolic acid B, 1.0 *μ*M, *n* = 5).

### 2.5. Radioimmunoassay for ANP

The concentration of ANP in perfusate was measured by a specific radioimmunoassay published previously [8]. Measurement of ANP was carried out using Tris-acetate buffer (0.1 M, pH 7.4; 0.2% neomycin, 1 mM EDTA, 50 BAEE units/mL SBTI, 0.002% sodium azide, and 1% bovine serum albumin). Bound and free form was separated with a charcoal suspension. The secreted amount of ANP was expressed as nanograms ANP per minute per gram of atrial wet weight.

### 2.6. Assay for Lactic Dehydrogenase Activity

Lactic dehydrogenase (LDH) activity was measured using a commercially available assay kit (Asan Pharmaceutical, Seoul, Korea) according to the manufacturer's instruction. Absorbance was measured at 340 nm in a microplate reader (Infinite F200 Pro, Tecan).

### 2.7. HPLC Fingerprinting and NMR Spectroscopy

**Figure 2:**
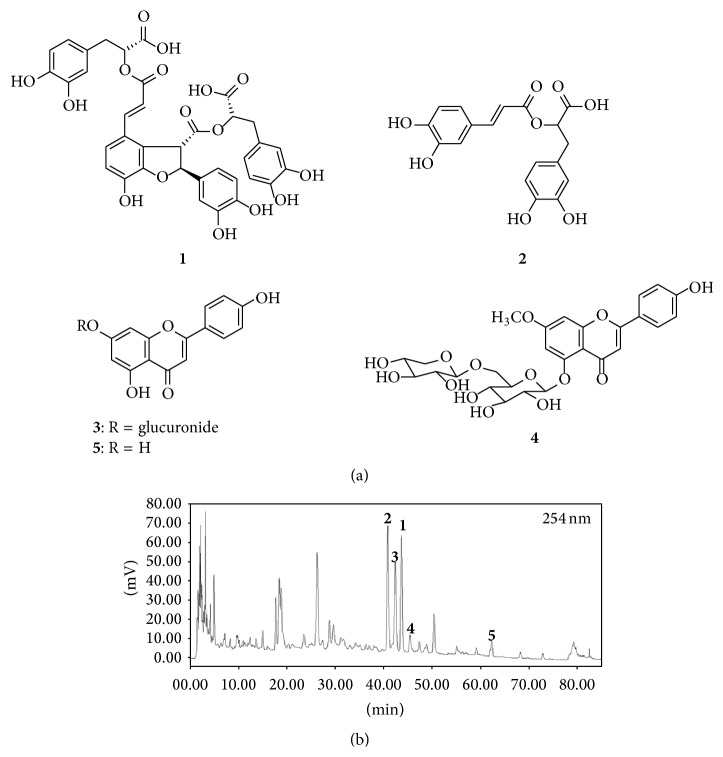
HPLC profile and H NMR analysis of Sibjotang. (a) Chemical structures of compounds. Number 1 is salvianolic acid B, number 2 is rosmarinic acid, number 3 is apigenin 7-O-b-glucuronide, number 4 is yuankanin, and number 5 is apigenin. It is analyzed by 1D- and 2D-NMR data; (b) HPLC chromatographic profile of the extract of SJ. Peak numbers correspond to structures given in (a), and the identity of the compounds was confirmed by retention times of isolated compounds.

The chromatographic fingerprint of SJ was performed on a YOUNGLIN system (YOUNGLIN Instrument, Korea) equipped with YOUNGLIN UV detector (UV 730D) and ZAM 3000 Evaporative Light Scattering Detector (Schambeck SFD GmbH, Bad Honnef, Germany). Chromatographic separation was carried out on a YMC C_18_ column (4.6 mm × 150 mm, 5 *μ*m) at room temperature with an injection volume of 30 *μ*L using a gradient elution of 10% methanol in water (0.1% formic acid) to 25% methanol in over 10 min and then to 50% methanol in water over 40 min, to 78% methanol in water over 25 min, and to 100% methanol over 1 min, followed by 100% methanol over 9 min. Peaks were detected simultaneously both at 210 nm and 254 nm of UV detection. The presence and identification of compounds were confirmed by comparisons of retention times of respective isolated compounds in the above analytical HPLC conditions (Figure 2(b)).

A portion of SJ (4.5 g) was then subjected to octadecyl functionalized silica gel flash column (75 *μ*m particle size 5 × 38 cm) chromatography. The column was eluted with a stepwise gradient of 20%, 50%, 80%, and 100% MeOH in H_2_O (four subfractions, 500 mL each), affording Fr. 1 to Fr. 4. The fraction eluted at 50% MeOH in H_2_O was then reapplied to octadecyl functionalized silica gel flash column (75 *μ*m particle size 5 × 20 cm) chromatography, eluting with a stepwise gradient of MeOH in H_2_O (from 10% to 80% with 10% increment, 8 fractions, 300 mL each). A portion (25.0 mg) of the fraction (78.1 mg) that eluted with 30% MeOH in H_2_O was further purified by semipreparative reversed-phase HPLC (Shiseido MG prep-C_18_ column (21.2 × 150 mm; 5 *μ*m particle size); 5 mL/min; 254 nm detection), eluting with a gradient from 10 to 100% MeOH in H_2_O (0.1% formic acid) over 60 min to yield a mixture of compounds** 1** and** 2** (*t*_*R*_ = 35.8 min) and** 3** (3.1 mg, *t*_*R*_ = 38.2 min). A mixture of compounds** 1** and** 2** was further purified by additional semipreparative reversed-phase HPLC (Agilent prep-C_18_ column (21.2 × 150 mm; 5 *μ*m particle size); 5 mL/min; 254 nm detection), eluting with a gradient from 40 to 65% MeOH in H_2_O (0.1% formic acid) over 40 min to afford compounds** 1** (3.2 mg, *t*_*R*_ = 20.3 min) and** 2** (1.0 mg, *t*_*R*_ = 21.7 min). A portion (36.5 mg) of the fraction (55.3 mg) that eluted with 80% MeOH in H_2_O from the first column chromatography was further purified by semipreparative reversed-phase HPLC (Shiseido MG prep-C_18_ column (21.2 × 150 mm; 5 *μ*m particle size); 5 mL/min; 254 nm detection), eluting with a gradient from 40 to 75% MeOH in H_2_O (0.1% formic acid) over 40 min to yield compounds** 4** (3.1 mg, *t*_*R*_ = 21.5 min) and** 5** (2.8 mg, *t*_*R*_ = 37.6 min).

NMR spectra (1D and 2D) were recorded using a JEOL JNM ECP-400 spectrometer (400 MHz for ^1^H and 100 MHz for ^13^C), and chemical shifts were referenced relative to the corresponding residual solvents signals. HSQC and HMBC experiments were optimized for ^1^ *J*_CH_ = 140 Hz and ^*n*^ *J*_CH_ = 8 Hz, respectively.

### 2.8. Statistical Analysis

The significance of the results was validated by one-way ANOVA with Bonferroni's multiple-comparison test and Student's *t*-test. *P* < 0.05 was considered as significant difference.

## 3. Results

### 3.1. HPLC Profile and H NMR Analysis

The structures of the compounds** 1–5** were identified as salvianolic acid B [14, 15], rosmarinic acid [16], apigenin 7-O-b-glucuronide, apigenin [17], and yuankanin [18], respectively, by analysis of 1D- and 2D-NMR data and comparisons with those reported (Figures 2(a) and 2(b)). The contents of the compounds** 1–5** in the extract were estimated as 0.2% (w/w), 0.06% (w/w), 0.2% (w/w), 0.1% (w/w), and 0.09% (w/w), respectively, on the basis of the isolation yields for the respective compounds.

### 3.2. SJ Increases ANP Secretion in a Concentration-Dependent Manner

**Figure 3:**
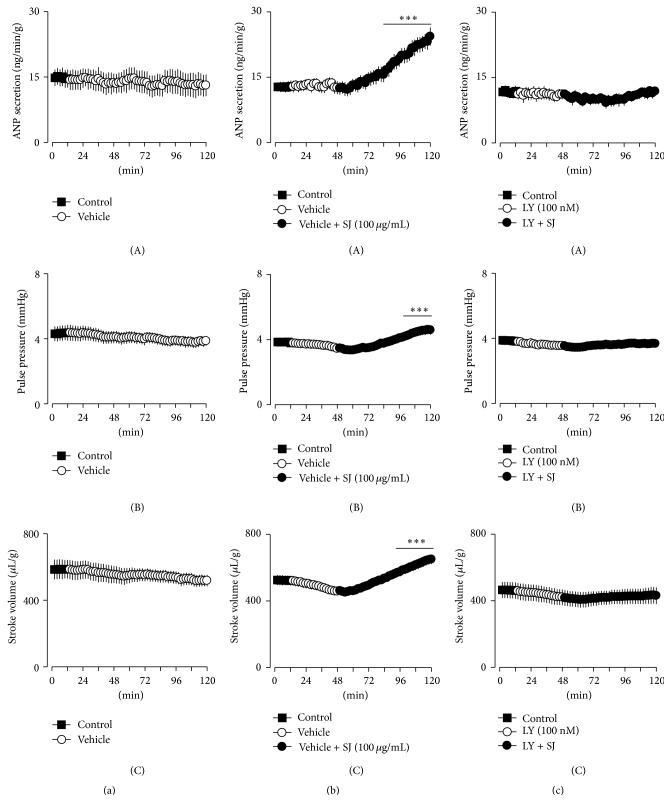
Effects of Sibjotang (SJ) on secretory and contractile function in perfused beating rabbit atria. (a) Effects of vehicle (DMSO 0.1%) on ANP secretion (A), pulse pressure (B), and stroke volume (C). (b) Effects of SJ (100 *μ*g/mL) on ANP secretion, pulse pressure, and stroke volume. (c) Effects of LY333531 (LY, 100 nM) on the SJ-induced increase in ANP secretion, pulse pressure, and stroke volume. Values are mean ± SE. Number of experiments: control, *n* = 8; SJ, *n* = 9; and LY + SJ, *n* = 8. ^*∗∗∗*^ *P* < 0.001 versus mean values of two fractions before SJ.

**Figure 4:**
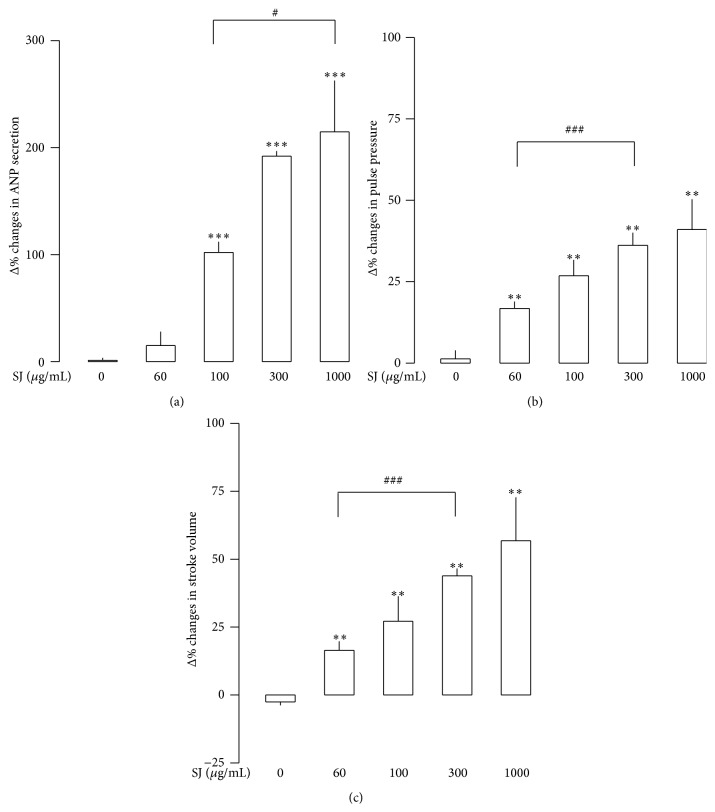
Concentration-dependent effects of Sibjotang (SJ) on ANP secretion, pulse pressure, and stroke volume in atria. (a) Effects of SJ on ANP secretion. (b) Effects of SJ on pulse pressure. (c) Effects of SJ on stroke volume. Values are mean ± SE. Number of experiments: SJ (0 *μ*g/mL, control), *n* = 6; SJ (60 *μ*g/mL), *n* = 8; SJ (100 *μ*g/mL), *n* = 8; SJ (300 *μ*g/mL), *n* = 5; and SJ (1,000 *μ*g/mL), *n* = 5. ^*∗∗*^ *P* < 0.01, ^*∗∗∗*^ *P* < 0.001 versus mean values of two fractions before SJ; ^#^ *P* < 0.05, ^###^ *P* < 0.001 versus control.

**Figure 5:**
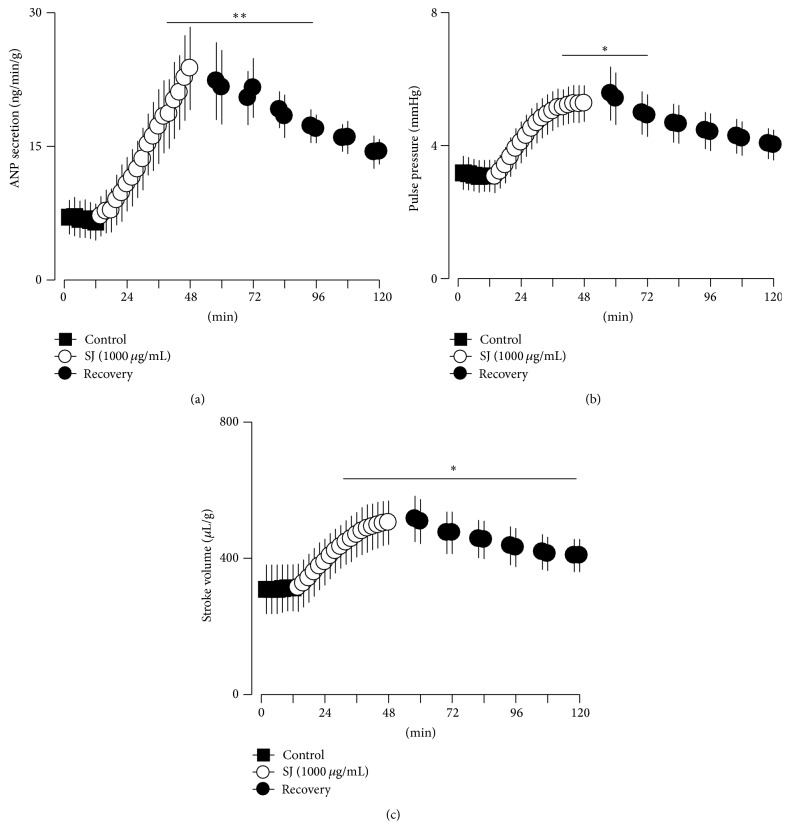
Recovery from the effects of Sibjotang (SJ) after replacing the buffer without the agent. (a) Recovery from the effects of SJ (1,000 *μ*g/mL) on ANP secretion. (b) Recovery from the effects of SJ on pulse pressure. (c) Recovery from the effects of SJ on stroke volume. Values are mean ± SE. Number of experiments, *n* = 4. ^*∗*^ *P* < 0.05, ^*∗∗*^ *P* < 0.01 versus mean values of two fractions before SJ.

Basal levels of ANP secretion remain steady and stable during experimental period in perfused beating atria (Figure 3(a)(A)). Similarly, atrial dynamics, pulse pressure, and stroke volume were stable (Figures 3(a)(B) and 3(a)(C)). Vehicle (vehicle for modulator, DMSO, 0.1%) had no significant effects on the basal levels of ANP secretion and atrial dynamics. SJ (100 *μ*g/mL) significantly increased ANP secretion, pulse pressure, and stroke volume (Figure 3(b)). SJ-induced increase in ANP secretion and positive inotropic effects were concentration-dependent (Figure 4). The increase in ANP secretion and atrial dynamics by SJ (1,000 *μ*g/mL) was persistent after replacing the buffer without the agent (Figure 5). The SJ-induced increase in ANP secretion was significantly higher than control up to 48 min after cessation of the drug (Figure 5(a)). Similarly, the SJ- (1,000 *μ*g/mL) induced positive inotropic effect remained elevated after the cessation of the drug (Figure 5(c)). In relation to the data of Figure 5, the levels of the LDH activity in perfusate showed no significant changes with SJ (100 *μ*g/mL) (data not shown). Higher concentration of SJ (1,000 *μ*g/mL) showed a transient but significant increase in the LDH activity in perfusate which was returned back to the control levels after cessation of the agent (3.93 ± 1.15% after 36 min of SJ, *P* < 0.05 versus control period; −1.06 ± 1.45% after 24 min of cessation of SJ, *P* > 0.05 versus control period). These findings indicated that SJ increased ANP secretion and atrial dynamics in a concentration-dependent manner.

### 3.3. SJ Increases ANP Secretion and Atrial Dynamics via PKC Pathway

**Figure 6:**
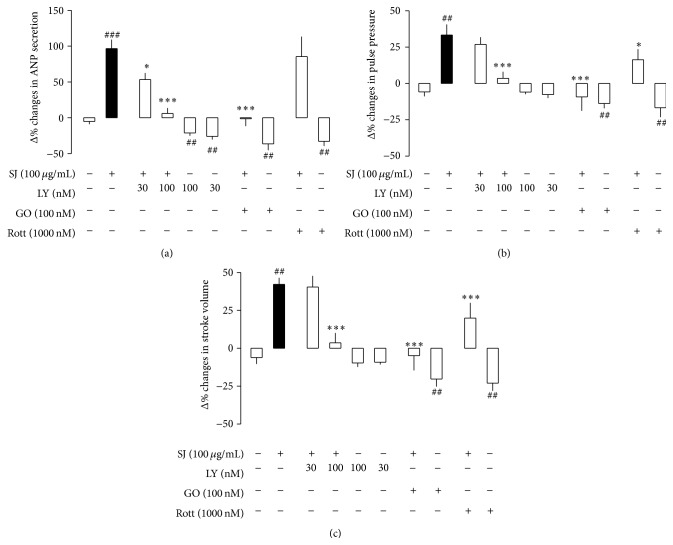
Effects of subtype-selective inhibitors of protein kinase C on the Sibjotang- (SJ-) induced increase in ANP secretion, pulse pressure, and stroke volume in atria. (a) Effects of inhibitors of protein kinase C on the SJ- (100 *μ*g/mL) induced increase in ANP secretion. (b) Effects of inhibitors of protein kinase C on the SJ-induced increase in pulse pressure. (c) Effects of inhibitors of protein kinase C on the SJ-induced increase in stroke volume. Values are mean ± SE. Number of experiments: control, *n* = 8; SJ (100 *μ*g/mL), *n* = 9; LY333531 (LY) 30 nM + SJ, *n* = 12; LY 30 nM + vehicle, *n* = 6; LY 100 nM + SJ, *n* = 8; LY 100 nM + vehicle, *n* = 5; GO6976 (GO) + SJ, *n* = 9; GO + vehicle, *n* = 5; Rottlerin (Rott) + SJ, *n* = 8; and Rott + vehicle, *n* = 6. ^*∗*^ *P* < 0.05, ^*∗∗∗*^ *P* < 0.001 versus SJ; ^##^ *P* < 0.01, ^###^ *P* < 0.001 versus control.

To identify the mechanisms involved in the SJ-induced changes in secretory and contractile function, effects of subtype-selective inhibitors of PKC were tested. LY, a selective inhibitor of PKC_*β*_, dose-dependently attenuated the SJ-induced increase in ANP secretion (Figures 3(c) and 6). LY (100 nM) completely inhibited SJ- (100 *μ*g/mL) induced increase in ANP secretion and positive inotropic effects. GO (100 nM), a selective inhibitor of conventional PKC_*α*_, inhibited SJ-induced changes in ANP secretion and atrial dynamics (Figure 6). However, Rott (1,000 nM), an inhibitor of novel PKC_*δ*_, had no significant effect on the SJ- (100 *μ*g/mL) induced increase in ANP secretion and arial dynamics (Figure 6). LY, GO, and Rott alone slightly but significantly decreased ANP secretion, and GO and Rott decreased atrial dynamics (Figure 6). These findings indicated that SJ increased ANP secretion and atrial dynamics via activation of conventional PKC signaling pathway.

### 3.4. Effects of Extract of Components Herbs and Ingredients of SJ

**Figure 7:**
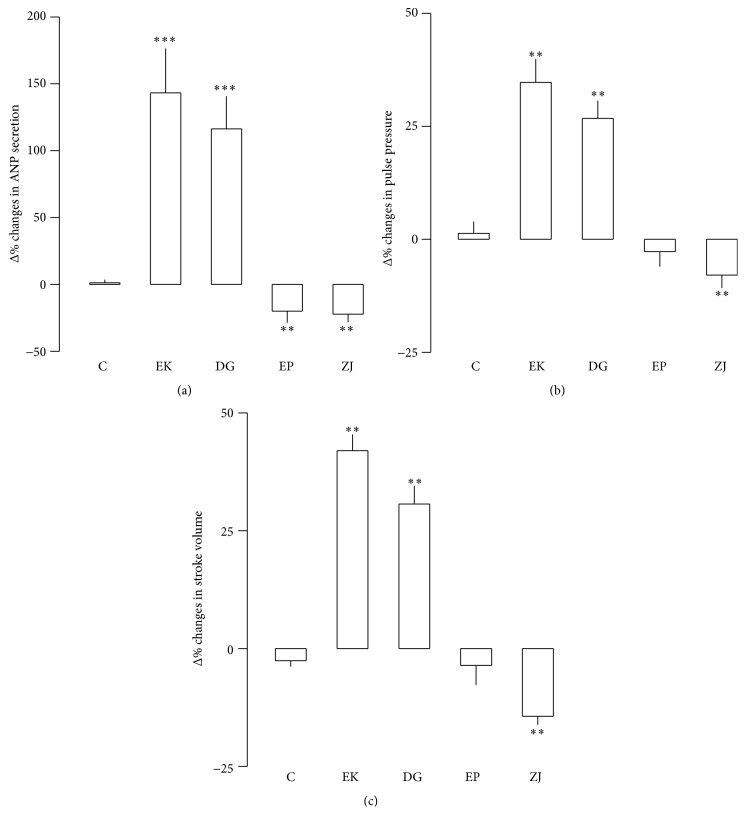
Effects of extract of component herbs of SJ,* Euphorbia kansui* (EK),* Euphorbia pekinensis* (EP),* Daphne genkwa* (DG), and* Ziziphus jujube* (ZJ) on ANP secretion, pulse pressure, and stroke volume in perfused beating rabbit atria. (a) Effects of extract of component herbs on ANP secretion. (b) Effects of extract of component herbs on pulse pressure. (c) Effects of extract of component herb on stroke volume. Values are mean ± SE. Number of experiments: control (C), *n* = 6; *n* = 7 for each of other groups. ^*∗∗*^ *P* < 0.01, ^*∗∗∗*^ *P* < 0.001 versus control.

To identify the roles of component herbs of the SJ, effects of four component herb extracts were separately tested. Extract of* Euphorbia kansui*, in the same concentration contained in SJ (1,000 *μ*g/mL), increased ANP secretion and atrial dynamics (Figure 7). Similarly,* Daphne genkwa* enhanced ANP secretion and atrial dynamics (Figure 7). However, extracts of* Euphorbia pekinensis* and* Ziziphus jujube* rather decreased ANP secretion (Figure 7(a)). Both extracts showed a negative inotropic effect (Figures 7(b) and 7(c)). To further identify the effects of chemical ingredients of the herbal components of SJ, experiments were performed. Because SJ contains apigenin, rosmarinic acid, and salvianolic acid B (Figure 2), experiments were performed to identify the effects of the ingredients on the ANP secretion and atrial dynamics. Apigenin (10 nM), rosmarinic acid (1.0 *μ*M), and salvianolic acid B (1.0 *μ*M) decreased ANP secretion by −23.34 ± 6.80%, −45.24 ± 8.20%, and −47.40 ± 5.28% (all *P* < 0.001 versus corresponding control group), respectively. Rosmarinic acid and salvianolic acid B but not apigenin decreased atrial pulse pressure by −7.93 ± 3.42% and −8.22 ± 1.48% (both *P* < 0.001 versus control group), respectively.

## 4. Discussion

The present study shows that a traditional herbal medicine SJ increases ANP secretion and atrial dynamics in perfused beating rabbit atria. The effects of SJ were concentration-dependent and persistent. SJ resulted in an increase in ANP secretion and positive inotropic effects via activation of conventional PKC subtypes. Conventional PKC_*α*_ and PKC_*β*_ inhibitors, GO and LY, respectively, but not novel PKC_*δ*_ inhibitor, Rott, attenuated SJ-induced increase in ANP secretion and atrial dynamics. In the regulation of ANP secretion from the heart, the involvement of PKC has been known. Previously, it was shown that phorbol ester, known to be an activator of PKC, accentuated the stretch-induced increase in ANP secretion from the atria [12, 19]. The present finding is consistent with the reports, furtherly suggesting the involvement of PKC_*α*_ and PKC_*β*_ signaling pathways in SJ-induced ANP secretion and atrial dynamics.

Extracts of two components of SJ,* Euphorbia kansui* and* Daphne genkwa*, increased ANP secretion and atrial dynamics similarly to SJ, while those of two other components slightly but significantly suppressed the secretory function. Although the roles of the latter two components are yet unknown, these findings suggest that* Euphorbia kansui* and* Daphne genkwa* are the main components in eliciting accentuation of cardiac hormone secretion and positive inotropic effect. The other two component herbs may conduct their role indirectly through modifying the effects of* Euphorbia kansui* and* Daphne genkwa*. Apigenin, rosmarinic acid, and salvianolic acid, chemical ingredients of SJ, decreased the atrial secretory and contractile function. Therefore, this latter finding suggests that apigenin, rosmarinic acid, and salvianolic acid B may not be directly involved in the atrial effects of SJ.

SJ-induced increase in ANP secretion may not be related to myocardial injury. The levels of LDH activity in perfusate were not significantly different between the values of before and after SJ (100 *μ*g/mL). A slight but transient increase in LDH levels during the infusion of higher concentration of SJ (1,000 *μ*g/mL) which has disappeared after cessation of the drug may be related to the increased washout of the enzyme in the extracellular space of the atrium because during this period atrial dynamics increased (Figure 4). Previously, it was shown that increase in atrial dynamics enhanced the extracellular fluid translocation in beating rabbit atria [8, 20].

The present study suggests that SJ could ameliorate impairment of the regulation of body fluid and blood pressure homeostasis via activation of the ANP system. Plasma levels of ANP are regulated by both the secretion from the atria and metabolism via degradation with neutral endopeptidase and clearance through nonguanylyl cyclase coupled natriuretic peptide receptor-C [21, 22]. Activation of ANP secretion induces an increase in the levels of plasma ANP and then urinary excretion of fluid and salt. The ANP system is also involved in the regulation of blood pressure via guanylyl cyclase-coupled natriuretic peptide receptor-A/B-cGMP signaling [1].

In conclusion, Sibjotang, traditional herbal medicine, induces an increase in ANP secretion and positive inotropic effects via activation of PKC signaling. The present study provides experimental evidence for the rationale in the use of SJ in the treatment of impairment of body fluid and blood pressure homeostasis.
